# Identificación de infección por el virus de la inmunodeficiencia humana en dos receptores de órgano sólido tres años después del trasplante

**DOI:** 10.7705/biomedica.7029

**Published:** 2024-08-29

**Authors:** Nancy Carolina Mopán, Diana Carolina Plazas, María Angélica Salinas, Yazmín Rocío Arias-Murillo, Jorge Alberto Cortés

**Affiliations:** 1 Grupo Red Donación y Trasplantes, Instituto Nacional de Salud, Bogotá, D.C., Colombia Instituto Nacional de Salud Instituto Nacional de Salud Bogotá, D.C. Colombia; 2 Departamento de Medicina Interna, Facultad de Medicina, Universidad Nacional de Colombia, Bogotá, D.C., Colombia Universidad Nacional de Colombia Universidad Nacional de Colombia Bogotá, D.C. Colombia

**Keywords:** donantes de tejidos, donantes de sangre, trasplante de órganos, trasplante de riñón, trasplante de hígado, seroconversión, VIH, transmisión de enfermedad infecciosa, biovigilancia, Tissue donors, blood donors, organ transplantation, kidney transplantation, liver transplantation, seroconversion, HIV, disease transmission, infectious, biosurveillance

## Abstract

El examen de rutina de los donantes de órganos para detectar la infección por el virus de la inmunodeficiencia humana (HIV) ha hecho que la transmisión del virus mediante el trasplante de órganos sea poco común. Sin embargo, a pesar de las pruebas de detección de rutina, la transmisión del HIV continúa siendo un riesgo del trasplante de órganos ya que, a diferencia de los tejidos, los órganos sólidos no se pueden procesar, desinfectar, ni modificar para inactivar patógenos infecciosos.

A continuación, se describe un caso de posible transmisión de HIV por trasplante de órganos de un donante previamente seronegativo a dos de sus receptores.

## Abstract (en)

Routine screening of organ donors to detect human immunodeficiency virus (HIV) infection has detected the rare transmission of the virus through organ transplantation. However, despite routine screening, HIV transmission remains a risk in organ transplantation since, unlike tissues, solid organs cannot be processed, disinfected, or modified to inactivate infectious pathogens.

A case of possible transmission of HIV by organ transplant is described below, from a previously seronegative donor to two recipients.

La sangre de los órganos de los donantes se elimina de forma rutinaria antes de su trasplante al receptor. Sin embargo, estos órganos aún pueden contener viriones del virus de la inmunodeficiencia humana (*Human Immunodeficiency Virus*, HIV) competentes para la replicación a partir de una pequeña cantidad de sangre del donante en el órgano -a pesar del lavado con soluciones de preservación- o de células infectadas del tejido ^1^.

Desde 1985, en Europa y en los Estados Unidos, a todos los donantes de sangre, tejidos y órganos se les practican pruebas rutinarias de detección de HIV, en las que se evalúan los anticuerpos contra el HIV mediante ELISA. No obstante, la detección de anticuerpos no excluye un riesgo de transmisión por donantes con infección por HIV (falsos negativos). Esto se debe a la existencia de un periodo -o ventana- de latencia inmunológica, durante el cual los anticuerpos contra el HIV aún no están presentes en el suero del donante ^1^. Este período varía entre dos y seis semanas después de la infección para la determinación de anticuerpos ^2^, aunque se ha informado que los viriones pueden estar presentes hasta durante 35 meses antes de la seroconversión. Después de la infección, el antígeno p24 se puede detectar hasta los 14 días y la carga viral se puede determinar a los cuatro a cinco días, o sea que la ventana inmunológica se puede acortar según la tecnología utilizada ^3^.

En Colombia, las pruebas para detectar el HIV se les practican a todos los donantes de componentes anatómicos, conforme a lo dispuesto en el Decreto 2493 de 2004 ^4^ y bajo las directrices establecidas por el Ministerio de Salud y Protección Social por medio del algoritmo de detección de la guía de práctica clínica para la atención de la infección por VIH/SIDA ^5^.

## Caso clínico

En octubre de 2021, una institución donde se practican trasplantes notificó al Sistema Nacional de Biovigilancia un caso sospechoso de transmisión de HIV por un trasplante hepático de donante fallecido a una paciente con diagnóstico de carcinoma hepático no diferenciado, procedimiento practicado en agosto del 2018. La paciente fue transfundida durante el trasplante y, posteriormente, recibió 10 transfusiones adicionales entre el 2018 y el 2021.

En el 2021, la paciente fue hospitalizada por posible rechazo del injerto hepático y se documentaron hallazgos clínicos indicativos de colangitis aguda; recibió tratamiento antibiótico con piperacilina-tazobactam por 10 días. Presentó detrimento en su evolución clínica, con falla hepática, por lo que se decidió iniciar el protocolo para nuevo trasplante y se tomaron pruebas infecciosas en las que se hallaron anticuerpos reactivos contra HIV. La prueba de *Western blot* confirmó el diagnóstico, se encontró una carga viral elevada (746.900 copias/ml) y un conteo disminuido de linfocitos T CD4+ (331 células/ml), con lo que se inició terapia antirretroviral.

En el momento del diagnóstico de la infección por HIV, habían transcurrido tres años y un mes desde el trasplante. Posteriormente, la paciente presentó múltiples complicaciones de su enfermedad de base, entre ellas, falla renal, por lo cual requirió una biopsia que se complicó con hemoperitoneo en el periodo posoperatorio. Se practicó una laparotomía de urgencia; sin embargo, presentó choque hipovolémico que no mejoró con el tratamiento y falleció 54 días después de haberse hecho el diagnóstico de HIV.

Inicialmente, el caso fue investigado por el Sistema de Información en Hemovigilancia (SIHEVI). Después de analizar los donantes de los componentes sanguíneos, se descartaron las transfusiones y, también, la vía sexual como fuentes de contagio. Una vez hecho esto, el SIHEVI trasladó el caso al Sistema Nacional de Biovigilancia para descartar la transmisión por el trasplante hepático.

Se planteó la hipótesis de que el donante de órganos hubiese estado en el período de latencia inmunológica y, por esta razón, se procedió a contactar a los demás receptores. A ellos se les hicieron pruebas de anticuerpos contra HIV de tipo I y II, las cuales resultaron positivas para el receptor del riñón izquierdo y, negativas, para los receptores del riñón derecho y las córneas (cuadro 1).

**Cuadro 1:** Seguimiento de los receptores tres años después del trasplante

Receptor	Componente anatómico trasplantado	Trasplante	Prueba para HIV	Prueba realizada	Resultado	Resultado
1	Hígado	Agosto de 2018	Agosto de 2021	Anticuerpos HIV I y II	Reactivo	Muerte por choque hemorrágico debido a hemoperitoneo detectado post mortem por biopsia renal
2	Riñón derecho	Agosto de 2018	Septiembre a octubre de 2021	Anticuerpos HIV I y II Carga viral	No reactivo No detectada	Sin rechazo del trasplante
3	Riñón izquierdo	Agosto de 2018	Octubre de 2021	ELISA DE cuarta generación para HIV I y II (electro quimioluminiscencia)	1^a^ y 2^a^ muestras reactivas	Muerte por vasculitis debida a HIV de origen embólico e infección fúngica diseminada
4	Córnea derecha	Agosto de 2018	Enero de 2022	HIV-1 / HIV-2 Anticuerpos totales (método inmunocromatográfico cualitativo)	No reactivo	Sin complicaciones reportadas
5	Córnea izquierda	Agosto de 2018	Marzo de 2022	ELISA	No reactivo	Rechazo de injerto

Fuente: Sistema Nacional de Biovigilancia, Instituto Nacional de Salud

El donante fallecido era un hombre sin antecedentes de importancia, excepto tabaquismo y consumo ocasional de alcohol. En la ficha institucional de validación del donante, se anotó la presencia de dos tatuajes en los miembros superiores de más de un año de realizados. El donante ingresó a urgencias en agosto del 2018 por una herida con arma de fuego en la región temporal derecha y tuvo tres paros cardíacos con reanimación exitosa. Requirió soporte múltiple y recibió una transfusión masiva de hemoderivados (cuatro unidades de glóbulos rojos y cuatro unidades de plasma) y antibióticos, y fue trasladado a la unidad de cuidados intensivos. Un día después del ingreso a urgencias, el donante evolucionó a muerte encefálica; se validó como donante efectivo, con perfil infeccioso negativo, incluyendo la prueba ELISA de tercera generación para detectar anticuerpos contra el HIV, por lo que se procedió al rescate del hígado, los riñones y las córneas. Los órganos fueron trasplantados horas después del rescate y, los tejidos oculares, a los ocho días.

En el curso de la investigación del caso adelantada por el Instituto Nacional de Salud, el SIHEVI rastreó y trazó las unidades de hemocomponentes transfundidas al donante. Se encontró que una de las unidades de plasma transfundidas al donante coincidía con un caso de transmisión de HIV previamente documentado y publicado en el *Boletín Seguridad Transfusional* por la coordinación de la Red Nacional de Bancos de Sangre y Servicios de Transfusión del Instituto Nacional de Salud ^6^.

El boletín reportaba una alerta emitida en febrero del mismo año, sobre la transmisión de HIV por la transfusión de una unidad de glóbulos rojos a una paciente de 13 años atendida en un centro de salud. Recibió 19 transfusiones entre el 5 de agosto y el 30 de noviembre del 2018. Mediante secuenciación genómica del virus en el donante y en el receptor, se logró identificar el mismo genotipo viral seis meses después del evento transfusional. El donante positivo fue remitido para tratamiento médico con antirretrovirales. Al mismo tiempo, se identificó un receptor adicional del mismo donante de sangre, quien recibió una unidad de plasma fresco congelado en agosto del 2018 durante un protocolo de reanimación cardio-cerebro-pulmonar y de transfusión masiva de hemoderivados. Este receptor falleció al día siguiente de la transfusión y en dicho boletín mencionaron que no había sido posible acceder a más información de la historia clínica por restricciones para su consulta, lo cual no le permitió al programa de hemovigilancia conocer que este receptor había sido donante de órganos y tejidos.

Finalmente, se confirmó que el receptor de la unidad de plasma fresco congelado había sido el donante de hígado de la paciente descrita y del receptor de riñón izquierdo, ambos reportados con infección por HIV con sospecha de transmisión por el trasplante. Sin embargo, no se practicaron pruebas para HIV, ni genotipificación viral del donante de los componentes anatómicos y los receptores, por indisponibilidad, insuficiencia o almacenamiento incorrecto de las muestras, debido al tiempo transcurrido entre la donación, el trasplante y la notificación del caso. Tampoco se hizo secuenciación genómica del HIV al receptor del riñón izquierdo para cotejarlo con el de la receptora del hígado, porque no hubo disponibilidad de muestras de plasma de los pacientes antes del inicio de la terapia antirretroviral.

### Consideraciones éticas

En el presente estudio, se utilizó información secundaria, datos de donantes y receptores que, en cumplimiento de la normatividad vigente, fueron ingresados al sistema de información RedDataINS. No se practicó ninguna intervención ni modificación intencionada de variables y, por tanto, la gestión realizada se catalogó como investigación sin riesgo, de acuerdo con la clasificación establecida en la Resolución 8430 de 1993 del Ministerio de Salud ^7^.

La investigación del caso se realizó en el marco del programa de biovigilancia ^8^, según lo establecido en la Resolución 2640 de 2005, en la cual se ordena la vigilancia epidemiológica de los trasplantes, “teniendo en cuenta los reportes hechos por los prestadores de servicios de salud en cuanto a reacciones adversas, complicaciones, rechazos y sobrevida” ^9^.

En el presente estudio, se protegió la confidencialidad de la información según las directrices establecidas y la normativa vigente de la Red Nacional de Donación y Trasplantes ^10^^,^^11^.

## Discusión

En este caso, se identificó un evento adverso grave de biovigilancia por posible transmisión de HIV de un donante cadavérico a dos de sus cinco receptores, específicamente, al receptor del riñón izquierdo y a la receptora del hígado. Se planteó la hipótesis de contagio de HIV por la transfusión, debido a que otra paciente que había recibido hemoderivados del mismo donante se infectó, lo cual se confirmó mediante la secuenciación genómica del virus. Diversas dificultades administrativas limitaron la posibilidad de que el sistema de vigilancia identificara de forma oportuna el riesgo de transmisión y los receptores infectados.

Los casos de transmisión de HIV de donante a receptor por medio de la trasfusión y el trasplante corresponden a un riesgo descrito en la literatura médica, basado en el concepto del período o ventana de latencia inmunológica del virus, que no se puede prever, y es uno de los riesgos que asumen los receptores al someterse a estos procedimientos. Los estudios realizados en los años 80 documentaron la posibilidad de la transmisión del virus a partir de los órganos trasplantados ^12^. Con la tecnología disponible en esa época, se reportó que los títulos de los anticuerpos contra el virus se podían diluir por efecto de transfusiones masivas, por lo que se recomendó la toma de muestras para pruebas de HIV antes de las transfusiones o varias horas después de las mismas, para evitar falsos negativos ^13^. El riesgo inicial documentado era muy alto, cercano al 85 %, y se evidenció en distintos tipos de trasplante de órganos sólidos ^14^. Esto llevó a distintas instituciones y a los *Centers for Disease Control and Prevention* (CDC) a implementar recomendaciones para el diagnóstico oportuno y la prevención de la transmisión de HIV, a principios de los años 90 ^15^.

El reconocimiento del trasplante como fuente de infección en los receptores infectados por HIV puede retrasarse porque se suele atribuir principalmente al uso de hemoderivados, pues los receptores de órganos a menudo reciben numerosas transfusiones de sangre y productos sanguíneos, y es mayor la probabilidad de transmisión del virus por vía sanguínea ^16^^,^^17^. En segundo lugar, los signos y síntomas de inmunosupresión pueden atribuirse a la terapia antirrechazo más que a la infección por HIV. Los receptores pueden morir a causa de enfermedades subyacentes antes de que se diagnostique la infección por HIV; de esta manera, la falta de reconocimiento y la comunicación en tiempo real del estado del receptor han sido características comunes de muchos casos de transmisiones de enfermedades derivadas del donante ^18^. En el caso de la receptora de hígado, se llegó al diagnóstico de forma incidental durante el protocolo de nuevo trasplante hepático por rechazo del injerto; y el receptor de riñón izquierdo fue diagnosticado a partir de las pruebas de laboratorio gestionadas durante el análisis del caso, pero previamente no se había sospechado que los receptores fueran portadores de HIV.

Los tiempos transcurridos en la identificación de los casos presentados reflejan la necesidad de integración de los sistemas de vigilancia, el cual debe fortalecerse para garantizar a los usuarios del sistema una mayor calidad y seguridad en el uso de componentes anatómicos.

Asimismo, los actores de la Red de Donación y Trasplantes deben tener en cuenta la posibilidad de transmisión de HIV por medio del trasplante de componentes anatómicos, por lo que es fundamental profundizar en la investigación de los antecedentes médicos, epidemiológicos y transfusionales durante la validación de los donantes, y hacer seguimientos periódicos después del trasplante a los receptores con este tipo de donantes. Estas acciones pueden contribuir a disminuir los casos de contagio y acortar los tiempos de diagnóstico de la infección. La evaluación cuidadosa de los antecedentes de un potencial donante para detectar riesgos asociados con infección por HIV y la exclusión de las personas que se encuentran en gran riesgo, aporta elementos clave para ayudar a excluir posibles donantes con infección reciente por HIV-1.

Según la literatura científica, deben considerarse factores asociados con un incremento del riesgo de un donante de contraer HIV, HBV o HCV ^19^. Sin embargo, la evaluación de los factores de riesgo de HIV-1 en donantes fallecidos suele ser compleja, ya que los familiares o amigos no siempre conocen la información completa y veraz del posible donante. La recomendación actual en países como Estados Unidos es la realización de una prueba molecular para detectar el virus ^20^.

Según la fisiopatología de la enfermedad, se sabe que una pequeña cantidad de sangre transfundida es suficiente para transmitir el HIV. La unión del virus a una célula CD4+ requiere, en promedio, de 30 minutos a dos horas, la retrotranscripción del ARN viral en ADN se completa después de aproximadamente seis horas y la integración en el genoma del huésped toma seis horas más. Aproximadamente 12 horas después de la integración, las primeras partículas del virus son detectables, es decir, los primeros virus se liberan de la célula infectada cerca de 24 horas después de la infección. La infección de HIV por medio de la sangre u órganos trasplantados, incluido el tejido óseo, puede ocurrir entre los días 5 y los 6 después de la transfusión o el trasplante ^21^.

Cuando se recibe una transfusión de sangre contaminada, el riesgo de transmisión es elevado; se ha calculado en el 89,5 %, pero, probablemente es más cercano al 100 % ^17^^,^^19^. Se ha demostrado que la concentración de HIV en la sangre es importante, pues en personas infectadas asintomáticas se encontró que una de cada 50.000 células mononucleares de sangre periférica estaba infectada, mientras que, en pacientes sintomáticos, la proporción fue una de cada 400 células ^22^. A pesar de que la sangre sea negativa para anticuerpos contra el HIV, aún existe riesgo, aunque extremadamente bajo, de desarrollar la infección; se ha estimado en uno por cada 153.000 unidades de sangre y en uno por cada 61.171 ^23^^,^^24^.

Durante la investigación del caso, se planteó la sospecha de infección del donante por medio de la transfusión, realizada un día antes de la extracción de órganos y tejidos, lo cual no coincide con el período reportado por la literatura para que el donante pudiera haber transmitido el virus a sus receptores. Por esta razón, se consideró también la posibilidad de que el donante ya se hubiese contagiado antes de la transfusión y que, al momento del rescate, se encontrara en un período de latencia inmunológica.

Sin embargo, es importante tener en cuenta que el riesgo estimado de transmisión del HIV por exposición sexual es inferior al reportado por transfusión sanguínea, según una revisión sistemática publicada por Patel *et al*. en AIDS en 2014 ^25^. Por otra parte, pueden presentarse falsos negativos en las pruebas para HIV, como en los pacientes con hemodilución secundaria a la transfusión masiva de hemocomponentes ^26^.

En este donante se verificó que las muestras usadas para las pruebas de infección se tomaron 33 horas antes de iniciar la transfusión, por lo que esta última hipótesis se descarta. A pesar de lo planteado, es viable la posibilidad de transmisión de HIV del donante a los receptores del riñón izquierdo y del hígado, con base en el antecedente de transfusión de plasma de un donante de sangre que presentó la misma variante viral de HIV que una de sus receptoras, lo cual se confirmó por secuenciación genómica del virus.

Se hizo una revisión bibliográfica en la biblioteca *Notify Library* (*The Global Vigilance and Surveillance Database for Medical Products of Human Origin*), donde se han reportado experiencias a nivel mundial de transmisión comprobada de HIV de donante a receptor por medio de trasplantes de órganos sólidos y tejidos. Muchos de estos casos se presentaron antes de 1985, cuando no se hacía tamizaje para HIV en los donantes; sin embargo, aún después de implantado el tamizaje rutinario para donantes de hemoderivados y otros componentes anatómicos, se han registrado casos de seroconversión, detectados entre los cinco días y los dos años y medio después del trasplante (cuadro 2).

**Cuadro 2:** Casos de transmisión de HIV por medio del trasplante

Autor	País	Año	Componente anatómico trasplantado	Señales de alerta, síntomas, evidencia de ocurrencia	Tiempo de detección	Resultado
Pirnay *et al*. ^1^	Bélgica	1997	Piel	El injerto de piel se utilizó antes de disponer del resultado serológico positivo del donante. Se observó una prueba de anticuerpos débilmente positiva en el receptor 37 días después de la exposición.	37 días	Sin datos clínicos de la evolución del paciente
Bowen *et al*. ^13^	EUA	1988	Riñón	El donante cadavérico de riñón recibió reposición masiva de hemoderivados. Se obtuvo una prueba negativa de anticuerpos contra HIV en	51 días	El receptor no presentó signos ni síntomas de infección por HIV después de un año.
Simonds *et al*. ^14^	EUA	1993	Riñón (n = 50), hígado (n = 13), corazón (n = 6), páncreas (n = 1), hueso (n = 4) y piel (n = 1)	En 61 casos, el trasplante se realizó antes de que comenzara la detección rutinaria de anticuerpos contra el HIV en los donantes	10-148 días	De 40 receptores con infección asociada con el trasplante de órganos a quienes se les realizó la prueba de anticuerpos contra HIV dentro de los seis meses posteriores al trasplante, 34 (85 %) fueron positivos; solo un receptor permaneció seronegativo más de seis meses después del trasplante.
Ison *et al*. ^18^	EUA	2011	Riñones, hígado y corazón	Diez meses después del trasplante de riñón derecho, se le practicó una biopsia renal que mostró rechazo celular agudo Banff 1A y glomerulonefritis proliferativa, posiblemente indicativa de infección por HIV.	8-10 meses	Dos pacientes fallecieron y los órganos trasplantados fallaron en los otros dos pacientes.
Borchi *et al*. ^27^	Italia	2010	Riñón	A los cuatro días después del trasplante los tejidos del donante fueron positivos para infección por HIV-1.	5 días	Buenas condiciones clínicas; los parámetros virológicos e inmunológicos se mantuvieron estables
Bellandi *et al*. ^28^	Italia	2010	Hígado y ambos riñones. El paciente también donó tejidos	Durante el proceso de donación, el resultado de la prueba de laboratorio se transcribió erróneamente de positivo a negativo, resultado que se incluyó en el registro de donación sin ninguna verificación, lo que conllevó la asignación del hígado y ambos riñones. El paciente también donó tejidos, por lo cual un segundo laboratorio evaluó su idoneidad. Cinco días después de los procedimientos de trasplante, el resultado positivo para HIV se remitió al Centro Regional de Trasplantes y se descubrió el error.	5 días	Los pacientes trasplantados fueron evaluados de inmediato y tratados con medicamentos antirretrovirales, con reporte de tratamiento exitoso y buenas condiciones generales en los tres pacientes a pesar de la transmisión de HIV por el trasplante de órganos
CDC ^29^	EUA	2011	Riñón	Primer caso confirmado de transmisión de HIV por trasplante de órganos de un donante vivo informado desde 1989; un año después del trasplante, el receptor fue hospitalizado con candidiasis oral y esofágica resistente; el cribado para infección por HIV mediante prueba inmunoenzimática fue positivo y la infección por HIV se confirmó con Western blot.	12 meses	Múltiples hospitalizaciones por enfermedad febril, episodios de insuficiencia renal y evaluación por posible rechazo del riñón trasplantado
Li *et al*. ^30^	Taiwán	2001	Hueso	A los cinco meses después del trasplante, la receptora se presentó en otro hospital con dolor e hinchazón en el muslo derecho. La prueba para detectar HIV fue positiva. No se informaron signos ni síntomas de infección clínica por HIV.	5 meses	No se proporcionaron detalles sobre el donante ni el receptor
Furlini *et al*. ^31^	Italia	1988	Médula	El análisis retrospectivo comenzó al descubrir que el donante era drogadicto (por vía intravenosa). El donante y el receptor fueron negativos para HIV antes del trasplante, pero hubo seroconversión cinco meses después	5 meses	Sin datos clínicos de la evolución del paciente
Anthuber *et al*. ^32^	Alemania	1991	Corazón	Un hombre de 38 años recibió un trasplante de corazón debido a una miocardiopatía dilatada en 1984, en un momento en que no se disponía de una prueba de detección para HIV. Ni el paciente ni el donante de órganos pertenecían a ninguno de los grupos de riesgo conocidos	2,5 años	En marzo de 1990 el paciente todavía no presentaba síntomas de sida.
Karcher *et al*. ^33^	Alemania	1997	Hueso	En enero de 1985, un hombre de 58 años con fractura de clavícula recibió un chip de hueso liofilizado trasplantado de un donante que abusaba de las drogas y que había muerto por sobredosis. El donante no se hizo la prueba para HIV y, en ese momento, solo había alrededor de 150 casos reportados de sida en Alemania.	Sin dato	El paciente desarrolló sida. Al menos, dos pacientes más de los 12 que recibieron trasplantes óseos del mismo donante, fallecieron más tarde a causa del sida.
Ahn *et al*. ^34^	EUA	2008	Hígado	Receptor y donante fueron seronegativos para HIV antes del trasplante. El donante, de orientación homosexual, recibió transfusión durante el rescate. Diez meses más tarde, se identificó mediante pruebas de ácidos nucleicos, HIV y VHC en el receptor y en los sueros almacenados del donante.	10 meses	El paciente desarrolló urosepsis, falla renal y hemorragias subdurales, y murió al año del trasplante.
Mukhopadhyay *et al*. ^35^	India	2012	Riñon	La receptora había recibido la donación de riñón de su hermana viva en octubre de 2007, y fue evaluada nueve meses después del trasplante por quejas de dismenorrea y periodos irregulares. Durante esta evaluación, se diagnosticó HIV; luego se confirmó el diagnóstico en su donante y en el esposo de la donante.	9 meses	El receptor inició tratamiento antirretroviral, tres semanas más tarde, desarrolló rechazo celular agudo y microangiopatía trombótica que mejoraron con esteroides y cambio de inmunosupresores. Después de esto, la creatinina se mantuvo estable (1,4 mg/dl).
Mitra *et al*. ^36^	India	2004	Riñón	En una repetición de la prueba para HIV durante su seguimiento posterior al trasplante, se reportó HIV-1 positivo por ELISA.	38 días	Después de una vida saludable de 39 meses después del trasplante, tuvo una breve enfermedad septicémica, con fiebre durante dos días y choque con síndrome de dificultad respiratoria aguda (SDRA), por lo que fue ingresada a UCI, falleció a las cuatro horas de la hospitalización.
Samuel *et al*. ^37^	EUA	1988	Riñón	A partir de los tres meses después del trasplante, el receptor desarrolló disfunción aguda del injerto, fiebre y candidiasis recurrente oral y esofágica. Aproximadamente un año después del trasplante, se descubrió que el receptor tenía infección por HIV	1 año	Once años después del trasplante, el receptor continuó teniendo un injerto funcional.

Fuente: Notify Library, the Global Vigilance and Surveillance Database for Medical Products of Human origin

La estandarización de la evaluación del riesgo de los donantes y el uso de pruebas moleculares en su selección, pueden ayudar a disminuir el riesgo de transmisión de enfermedades por medio del trasplante de órganos de un donante serológicamente negativo ^38^. Sin embargo, la conveniencia de utilizar técnicas moleculares en el tamizaje de los donantes es discutida pues, a pesar de ser una técnica muy sensible y avanzada, que ha reducido el período de latencia inmunológica del HIV a 2,93 días, en el contexto colombiano implica grandes costos, baja disponibilidad y limitaciones por la prolongación de tiempos para obtener el resultado ^39^.

Una alternativa para los laboratorios de las instituciones donde se practican los trasplantes es optar, en lo posible, por pruebas ELISA de cuarta generación para detectar anticuerpos contra el HIV, debido al reducido tamaño del período de latencia inmunológica que brindan estas pruebas en comparación con las de tercera generación ^40^.

## Conclusión

Se confirmó la presencia de HIV en receptores de órganos sólidos en un tiempo mayor de tres años después del trasplante, en quienes se descartó la transmisión por vía transfusional o sexual, y se consideró como vía de contagio el órgano donado. El papel del Sistema de Biovigilancia es fundamental en los procesos de la Red de Donación y Trasplantes en la medida que permite mediante la trazabilidad de los componentes anatómicos agilizar la identificación de receptores infectados para evitar la distribución de componentes anatómicos no utilizados y proceder a su descarte. Además, favorece el inicio temprano del tratamiento de la infección y la prevención de la transmisión del virus a otros individuos por los receptores infectados. Por esta razón, se debe fortalecer la integración con los demás sistemas de vigilancia mediante el aumento de la calidad y la seguridad en los procesos, y la mitigación de los efectos de eventos adversos e incidentes que puedan ocurrir en todos los eslabones de la cadena, desde la donación hasta el trasplante.

En el contexto de la selección de donantes de órganos y tejidos, es crucial destacar la importancia de incluir la estandarización de la evaluación del riesgo de los donantes de órganos en guías y protocolos nacionales, donde se consideren las pruebas de última generación para detectar el HIV como parte del tamizaje o plantear la inclusión de pruebas moleculares, a fin de disminuir los riesgos asociados con la trasmisión de este tipo de virus. Asimismo, es imperativa la actualización de la normativa sobre los procesos de donación y trasplantes, y que temas tales como los perfiles infecciosos dependan de directrices técnicas que puedan modificarse rápidamente según el avance tecnológico en salud o la disponibilidad de pruebas en el país.

El costo adicional de las pruebas moleculares para HIV resulta insignificante en comparación con los costos totales del trasplante. Esta medida conlleva beneficios significativos al prevenir posibles riesgos derivados de la falta de detección del virus en los donantes. La omisión de estas pruebas podría resultar en la transmisión inadvertida del virus a los receptores de órganos o tejidos trasplantados. La detección temprana y precisa del HIV en los donantes es crucial para garantizar la seguridad y eficacia de los trasplantes, protegiendo la salud y el bienestar de los receptores y evitando, así, complicaciones potenciales y costos adicionales asociados con la transmisión no detectada del virus.

Teniendo en cuenta las limitaciones presentadas en el presente caso, al no poder establecerse la asociación causal definitiva por falta de muestras o por muestras insuficientes para el procesamiento, es necesario trabajar en la implementación de un banco de almacenamiento de muestras sanguíneas de donantes de órganos por parte de la entidad rectora en salud, para poder disponer de ellas en casos que requieran la trazabilidad de los componentes anatómicos relacionados con incidentes graves o eventos adversos.
